# Treatment-seeking rates in malaria endemic countries

**DOI:** 10.1186/s12936-015-1048-x

**Published:** 2016-01-11

**Authors:** Katherine E. Battle, Donal Bisanzio, Harry S. Gibson, Samir Bhatt, Ewan Cameron, Daniel J. Weiss, Bonnie Mappin, Ursula Dalrymple, Rosalind E. Howes, Simon I. Hay, Peter W. Gething

**Affiliations:** Spatial Ecology and Epidemiology Group, Tinbergen Building, Department of Zoology, University of Oxford, South Parks Road, Oxford, UK; Wellcome Trust Centre for Human Genetics, University of Oxford, Roosevelt Drive, Oxford, UK; Fogarty International Center, National Institutes of Health, Bethesda, MD USA; Institute for Health Metrics and Evaluation, University of Washington, Seattle, WA USA

**Keywords:** Malaria, Fever, Treatment-seeking, Care-seeking, Modelling, *Plasmodium vivax*, *Plasmodium falciparum*

## Abstract

**Background:**

The proportion of individuals who seek treatment for fever is an important quantity in understanding access to and use of health systems, as well as for interpreting data on disease incidence from routine surveillance systems. For many malaria endemic countries (MECs), treatment-seeking information is available from national household surveys. The aim of this paper was to assemble sub-national estimates of treatment-seeking behaviours and to predict national treatment-seeking measures for all MECs lacking household survey data.

**Methods:**

Data on treatment seeking for fever were obtained from Demographic and Health Surveys, Malaria Indicator Surveys and Multiple Cluster Indicator Surveys for every MEC and year that data were available. National-level social, economic and health-related variables were gathered from the World Bank as putative covariates of treatment-seeking rates. A generalized additive mixed model (GAMM) was used to estimate treatment-seeking behaviours for countries where survey data were unavailable. Two separate models were developed to predict the proportion of fever cases that would seek treatment at (1) a public health facility or (2) from any kind of treatment provider.

**Results:**

Treatment-seeking data were available for 74 MECs and modelled for the remaining 24. GAMMs found that the percentage of pregnant women receiving prenatal care, vaccination rates, education level, government health expenditure, and GDP growth were important predictors for both categories of treatment-seeking outcomes. Treatment-seeking rates, which varied both within and among regions, revealed that public facilities were not always the primary facility type used.

**Conclusions:**

Estimates of treatment-seeking rates show how health services are utilized and help correct reported malaria case numbers to obtain more accurate measures of disease burden. The assembled and modelled data demonstrated that while treatment-seeking rates have overall increased over time, access remains low in some malaria endemic regions and utilization of government services is in some areas limited.

**Electronic supplementary material:**

The online version of this article (doi:10.1186/s12936-015-1048-x) contains supplementary material, which is available to authorized users.

## Background

Although millions are affected by clinical malaria each year, the last 15 years have seen unprecedented gains from international efforts made to control this disease. The establishment of the Roll Back Malaria initiative and the Millennium Development Goals in the year 2000 were followed by a nearly 20-fold increase in international funding for malaria control [1]. The scale-up of interventions that followed has resulted in a 40 % decline in *Plasmodium falciparum* clinical incidence in Africa while prevalence of the infection has nearly halved since the year 2000 [2]. This and marked reductions in malaria-associated deaths [3] were largely attributed to increased coverage of the insecticide-treated bed nets (ITNs). Second to ITNs, which were the most widespread intervention, access to artemisinin-based combination therapy (ACTs) has been found to greatly impact the incidence of disease [2].

Prompt diagnosis and treatment of clinical malaria is the mainstay of all control or elimination programs [4–6]. The primary aim of treatment with ACT is to curtail clinical disease in patients, though access to effective treatment also impacts onward transmission to the wider community by reducing the infectious reservoir while at the same time containing the spread of drug resistance [2]. Treatment coverage has been assessed across Africa [7], but understanding how access to treatment varies throughout the malaria endemic world is essential to evaluating the true public health impact of treatment coverage. This is all the more significant during the current transition from control of clinical disease towards regional focuses on malaria elimination and the post-2015 future is shaped as progress towards the Millennium Development Goals is evaluated [8].

In assessing care-seeking behaviours, it is also important to consider what proportion of care is sought at government-based facilities. Government facilities are more likely to comply with recommended diagnostic and treatment schedules [1, 9], and to have their routine records integrated into the national health data management system. Even in areas with strong surveillance systems, reports of passively detected malaria cases will capture only a certain fraction of all malaria cases [10–13] and so must be adjusted by a number of parameters before use as official burden estimates [1, 14]. These include (1) treatment-seeking behaviour (representing the proportion of cases not attending health facilities and thus being omitted from aggregated case reports [15–22] as well as the proportion seeking treatment outside the public health system); (2) malaria diagnoses made presumptively without parasitological confirmation (leading to reported case numbers including non-malaria illnesses [23, 24]); and (3) incomplete reporting (which leads to cases being lost from reported data) [1, 25]. Here we aim to improve the evidence-base of the first parameter to enable refined estimates of the true clinical burden of malaria disease.

Treatment-seeking rates vary widely between countries and greatly affect final burden estimates. Where available, these parameters are drawn from nationally representative, cross-sectional, household surveys such as Demographic and Health Surveys (DHS) [26], Malaria Indicator Surveys (MIS) [27] and UNICEF Multiple Indicator Cluster Surveys (MICS) [28]. However, not all malaria-endemic countries (MECs) have such survey data available, resulting in these important parameters being either assumed or omitted and making comparisons of access to treatment across all malaria endemic regions and effective estimation of the burden of disease difficult.

The aim of this study was an exhaustive assembly of the available data on treatment-seeking for all MECs, sub-nationally where possible. For MECs lacking national survey data, predictive models were built to estimate use of public facility treatment as well as treatment of any kind. These estimates, including measurements of their uncertainty, will allow for improved understanding of how health services in endemic countries are routinely accessed and facilitate more accurate disease burden estimation.

## Methods

### Data assembly

Many DHS, MIS and MICS ask questions to determine the prevalence and treatment of fever in children less than 5 years of age. Here it was assumed that the treatment-seeking rates observed in children would be similar in older age groups, as supported by evidence from India, Indonesia and Ethiopia [16, 25]. For a handful of DHS surveys (*n* = 4) and earlier MICS rounds (MICS 3 and 4, *n* = 45), data on treatment-seeking for fever were not available, so data on treatment-seeking for acute respiratory infection (ARI) were used, which have previously been shown to correlate strongly with treatment-seeking for fever [25]. For surveys that contained treatment-seeking data, the survey codes for questions regarding where treatment was sought were reviewed and categorized into public/government facilities (likely to have been captured by reporting systems) or ‘any’ medical treatment, which included private or NGO facilities, but excluded non-medical categories of care such as homeopathic doctors or ‘healers’. To generate a comprehensive database of treatment-seeking information, no time restriction was placed on the year of the survey. However, only MICS conducted in the third survey round or later (from 2005) were included due to inconsistency in interview questions on where treatment was sought within earlier surveys.

From each survey containing data on fever (or ARI) treatment-seeking, the total number of children reported to have fever was summarized by cluster and region. The numbers seeking treatment at either category of facility were also totalled to obtain the proportion of those who seek public/government facility-based treatment or any treatment. These data extractions from the DHS and MICS websites [26, 28] and summaries were all automated using Feature Manipulation Engine (FME) version 2015 by Safe Software [29]. MIS data were extracted from the DHS platform and will be discussed as part of the DHS survey from here onward. The total number of children, fever cases and cases that sought treatment were then summarized nationally in order to generate predictions for those countries where survey data were unavailable. National survey data require the incorporation of sampling weights, which are provided in the survey results and help to adjust for differences in the probability of selection to produce a more accurate representation of population-based metrics. Sampling weights were extracted and applied at the individual level following DHS guidelines [30], such that the totals and proportions reported here were consistent with the same metrics (percentage of fever or illness) provided in the official survey reports.

### Covariate data

Potential covariates were identified for inclusion in a predictive model using a review of the literature for ‘treatment-seeking’ and ‘care-seeking’ for both ‘malaria’ and ‘fever’ in PubMed on 18 July 2015 [31]. The following have been reported as determinants of care-seeking rates: household wealth [15, 17, 19, 20, 22, 32–38], care-giver education [15, 19, 32, 34, 39] and household location (rural or urban) or access to health facilities [15, 20–22, 32, 33, 40–45]. The World Bank provides freely available national-level indicator data [46], and several indicators that were in keeping with the themes identified by the literature were downloaded: access to electricity (as a proxy for wealth and access to health facilities), gross domestic product (GDP; current US$), GDP per capita, GDP growth (annual %), gross national income (GNI) per capita (current US$), total health expenditure (% of GDP), public health expenditure (% of total), primary education completion rate (% of relevant age group), and rural population (% of total population). In addition to these metrics, the number of health workers was thought to influence treatment-seeking rates. However, there were not sufficient data on the number of community health workers per 1000 people available from the World Bank, so the number of nurses and midwives per 1000 population was used instead. Finally, explicit care-seeking variables were also included: the percentage of pregnant women receiving prenatal care and the percentage of children aged 12–23 months who were immunized against diphtheria, pertussis and tetanus (DPT).

Matching covariate data to national-level survey data was also achieved using FME. When available, covariate data were used from the same year as the survey, and otherwise were matched to the closest year. Covariate data were also matched to those countries without treatment data available. For these countries, covariate data was matched to 2013, which was the most recent year of indicator data available, or the closest year to that in order to generate the most up to date estimates of treatment-seeking rates.

**Fig. 1:**
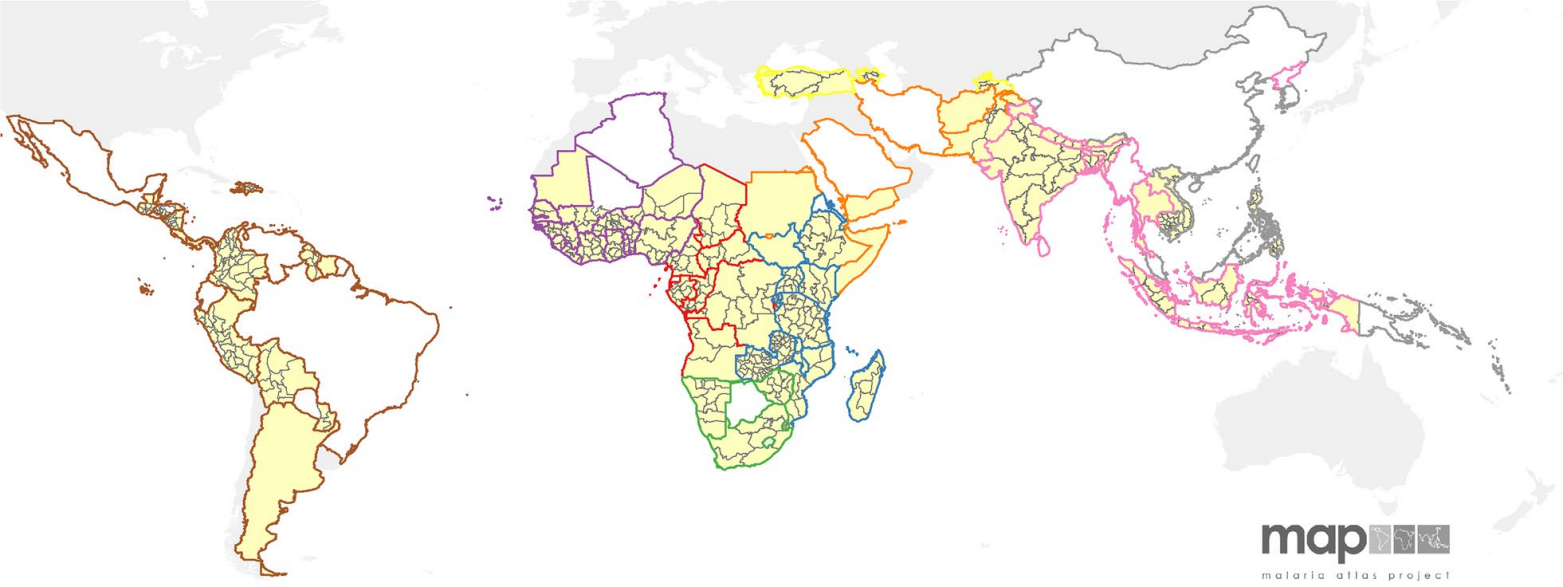
Treatment-seeking data in malaria-endemic countries by WHO region. MECs with treatment-seeking data available are *shaded yellow* and those missing data are shown in *white*. Country borders are coloured based on WHO region: Central Africa (AFRO-C, *red*), East Africa and high-transmission areas in Southern Africa (AFRO-E, *blue*), low-transmission Southern African countries (AFRO-S, *green*) West Africa (AFRO-W, *purple*), Americas (PAHO, *brown*), Eastern Mediterranean (EMRO, *orange*), Europe (EURO, *yellow*), Southeast Asia (SEARO, *pink*) and Western Pacific (WPRO, *grey*). Areas shaded *grey* outside the coloured borders have no malaria risk

In addition to the social and economic covariate data, countries were grouped geographically based on their WHO regional offices: Region of the Americas (PAHO), Eastern Mediterranean Region (EMRO), European Region (EURO), Southeast Asia Region (SEARO), and Western Pacific Region (WPRO). Countries in the African region were separated into the sub-African regions reported in the World Malaria Report: West Africa (AFRO-W), Central Africa (AFRO-C), East Africa and high-transmission areas in Southern Africa (AFRO-E), and low-transmission Southern African countries (AFRO-S) [1]. These regions then formed strata within the model, as explained below. A map of georeferenced treatment-seeking data availability from national surveys in MECs is shown in Fig. 1.

### Statistical modelling

Two generalized additive mixed models (GAMMs) were developed to predict the proportion of patients that sought treatment from (1) a facility covered by the government reporting system, and, (2) any type of medical care provider [47, 48]. Where multiple surveys were used from the same country, this was accounted for by adding a country-level random effects term. Both year and WHO region were included as fixed effect terms, along with the suite of country-level covariates. The list of the covariates described above was limited after testing for collinearity among all the potential variables. The variables included in GAMMs were: year of survey, WHO region, GDP growth, health expenditure, prenatal care rates, primary education, DPT immunization rates, nurses and midwives per population and proportion rural population. All statistical analyses were performed in the R statistical computing environment [49]. Full details of the model development are provided in Additional file 1.

### Mapping treatment-seeking

Existing treatment-seeking data were mapped at the regional level (boundaries shown in Fig. 1) for the 76 countries with available survey data. Although some of these data were available at a point level, only regional maps were generated for consistency with the other adjustment parameters. For the few countries with DHS cluster data and no regional data available, cluster level data were summarized and mapped to the first- (Colombia) or second-level (Zambia) administrative units [50]. Predicted and observed values were then combined into a single, geographically complete, map of treatment-seeking for all MECs showing the most recent available data in each country.

## Results

### Data assembly

Treatment-seeking data were collected from DHS (*n* = 195, 13 of which were MIS) and MICS (*n* = 59) for 76 countries. The number and year of surveys available from MECs are shown in Additional file 1: Figure S1. Data from DHS and MICS round 5 collected data on treatment-seeking for fever, whereas MICS rounds 3 and 4 assess care-seeking for cough (respiratory infection). Additional file 1: Figure S2 confirms that rates observed from the earlier MICS rounds are comparable to the treatment-seeking for subsequent surveys. Indicator data were available for all but nine surveys (there was no health expenditure data available for Zimbabwe and Somalia); these nine surveys could not therefore be included in the analysis. DHS surveys conducted prior to 1990 were excluded as they reported that no individuals sought treatment, and were therefore considered to be non-representative. Two MICS surveys, Yemen (2006) and Burkina Faso (2006), also reported zero treatment-seeking and were excluded. The GAMMs were thus fitted to 228 records from 72 countries. This left 22 MECs for which predictions of treatment-seeking outcomes were required. However, Brazil and Paraguay were also added to the prediction list since Brazil only had national level data available (DHS 1996) and Paraguay had large areas of missing data (DHS 1990). This increased the number of prediction countries to 24.

### Model results

Two models were chosen as the best candidate models for treatment-seeking from public facilities and five for seeking any treatment. The fitted coefficients, AIC, ΔAIC and ω_i_ values for each of the best models are shown in Table 1. Model average coefficient values for each geographic region are shown in Additional file 1: Table S1. The parameter with greatest model influence was the percentage of pregnant women receiving prenatal care, followed by DPT immunization rates for pubic treatment-seeking and primary education completion for any treatment. Geographic regions were also statistically significant, as was public health expenditure. Time was also included as an indicator variable because all available surveys with treatment-seeking information were included (1990 and 2005 onwards for DHS and MICS, respectively. Plots of observed treatment-seeking rates over time (see Additional file 1: Figure S3) confirmed that rates of treatment-seeking from any type of facility and particularly from government-run facilities increased over time. Model validation showed good prediction performance (Additional file 1: Figures S4–S6). The root mean square error (RMSE) for the percentage seeking treatment from government facilities was 11.9 and 12.3 % for any treatment facility type.

**Table 1:**
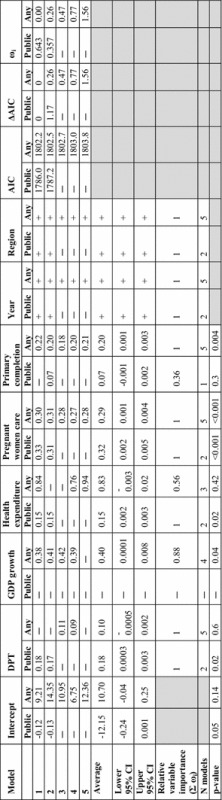
**Average generalized additive mixed model (GAMM) coefficients, 95 % CIs, selection criteria and relative variable contributions. AIC is the Akaike information criterion, ΔAIC is difference in AIC and ω**_**i**_ **is the Akaike weight**

All observed and predicted values, as well as covariate data, are shown in Additional file 2. There were five countries for which insufficient covariate data precluded predictions. These countries were therefore assigned estimates from a similar and ideally neighbouring country in the same region (rounded to two decimal places to show that they are less precise). Myanmar (Burma) was matched to Timor-Leste, which had the most similar health indicator variables (DPT and pregnant women care). In the absence of indicator data or neighbouring countries in the same WHO region the Democratic People’s Republic of Korea (DPRK) was given the same estimate as Myanmar (Burma). Eritrea was given the same value of neighbouring Ethiopia, French Guiana was given Suriname’s values, and the Republic of Korea was given China’s estimate, which was closest to Korea for the indicators that were available.

**Fig. 2:**
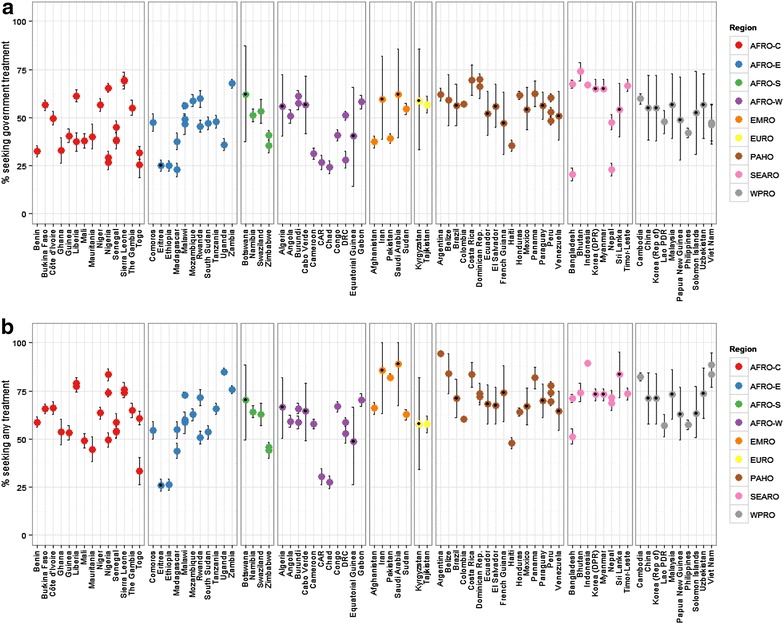
Predicted and observed treatment-seeking proportions. The predicted proportions estimated for 2013 are shown alongside observed values from 2010 onwards for government treatment-seeking (**a**) and treatment-seeking from any facility (**b**). Points are coloured by WHO region and shown with 95 % CI *error bars*. Predicted estimates are overlaid with *black points*. *Each point* represents a spatial aggregate of a single national survey. Countries with multiple points indicate multiple survey types or years

The mean predicted values for the proportion seeking treatment (government-based or other) in the 24 countries without survey data available are shown alongside the post-2010 observed treatment-seeking values in Fig. 2. The 95 % CI of the observed measures were obtained from the standard error of national weighted mean. The predicted measures are shown with black points, but are also distinguishable by wider uncertainty resulting from the model predictions being made from sampling within the confidence intervals of the observed measures. In spite of their greater uncertainty, the predicted point estimates follow the pattern of the observed estimates in each region. These plots reveal some areas where the proportion of treatment-seeking from government-based facilities is markedly lower, notably in SEARO. Greater heterogeneity in estimates of both types of treatment-seeking was observed among the countries in the African regions.

Despite variability between geographic regions, treatment-seeking rates have generally increased over time. The non-linear relationship between treatment-seeking and time is shown in Additional file 1: Figure S3. The lowest rates of access to government treatment (<10 %) were observed prior to the year 2000, with the exception of findings from DHS surveys from Chad (2004), Bangladesh (2004 and 2007) and Pakistan (2007) and a MICS from Somalia in 2006 which were all also <10 %. The lowest reported rate of seeking any treatment was 24 % in Togo in 2006. All observations of treatment-seeking rates less than 40 % (from any health facility) were from the African continent (including Somalia, from EMRO). The highest measures of access to public facilities (>65 %) were observed in several regions: AFRO-C (Nigeria and Sierra Leone), AFRO-E (Zambia and Mozambique), PAHO (Costa Rica, Dominica Republic and Peru) and SEARO (Bangladesh (in 2011, but not in the earlier years referenced above), Bhutan, Indonesia and Timor-Leste). Bhutan had the highest reported measure of 74 % of individuals seeking care at government-based facilities (MICS 2010). High rates of access to any treatment type likewise spanned various regions, but values were far greater than public-facility data only. Regions with observations of access rates to any treatment greater than 80 % include: AFRO-C (Nigeria), AFRO-E (Tanzania and Uganda), EMRO (Pakistan), PAHO (Argentina, Belize, Costa Rica, Dominican Republic and Panama), SEARO (Indonesia (since 1991) and Thailand) and WPRO (Cambodia and Viet Nam).

Regional comparisons of treatment-seeking rates are shown in Table 2. Weighted estimates were obtained by multiplying the most recent observed or predicted value for each of the 98 MECs by the proportion of the regional population in each country. Population data from 2013 [51] were summarized nationally in ArcGIS [52]. While SEARO has lowest overall government-based treatment-seeking behaviours, this region also has the highest access to any treatment. Patterns within the other regions were not as marked, but access to treatment including facilities outside the formal government sector was overall higher.

**Table 2:** Weighted means of treatment-seeking rates by WHO region

WHO region	Government treatment			Any treatment		
	Mean %	Upper %	Lower %	Mean %	Upper %	Lower %
AFRO-C	45.39	49.14	41.63	63.08	66.76	59.40
AFRO-E	40.26	43.59	36.93	56.29	59.31	53.27
AFRO-S	46.69	50.36	43.04	69.19	72.38	65.94
AFRO-W	48.91	54.52	43.52	59.79	65.12	54.32
EMRO	46.30	57.34	35.86	71.95	79.54	61.10
EURO	52.91	58.76	47.16	56.08	61.90	50.29
PAHO	55.13	61.98	48.59	71.84	77.67	66.05
SEARO	27.62	29.13	26.25	78.83	80.23	77.42
WPRO	54.13	70.52	37.87	71.41	84.02	58.94

The most recent treatment-seeking values for all 98 MECs were weighted by size of the population to obtain weighted point, upper and lower estimates

### Mapping treatment-seeking

**Fig. 3:**
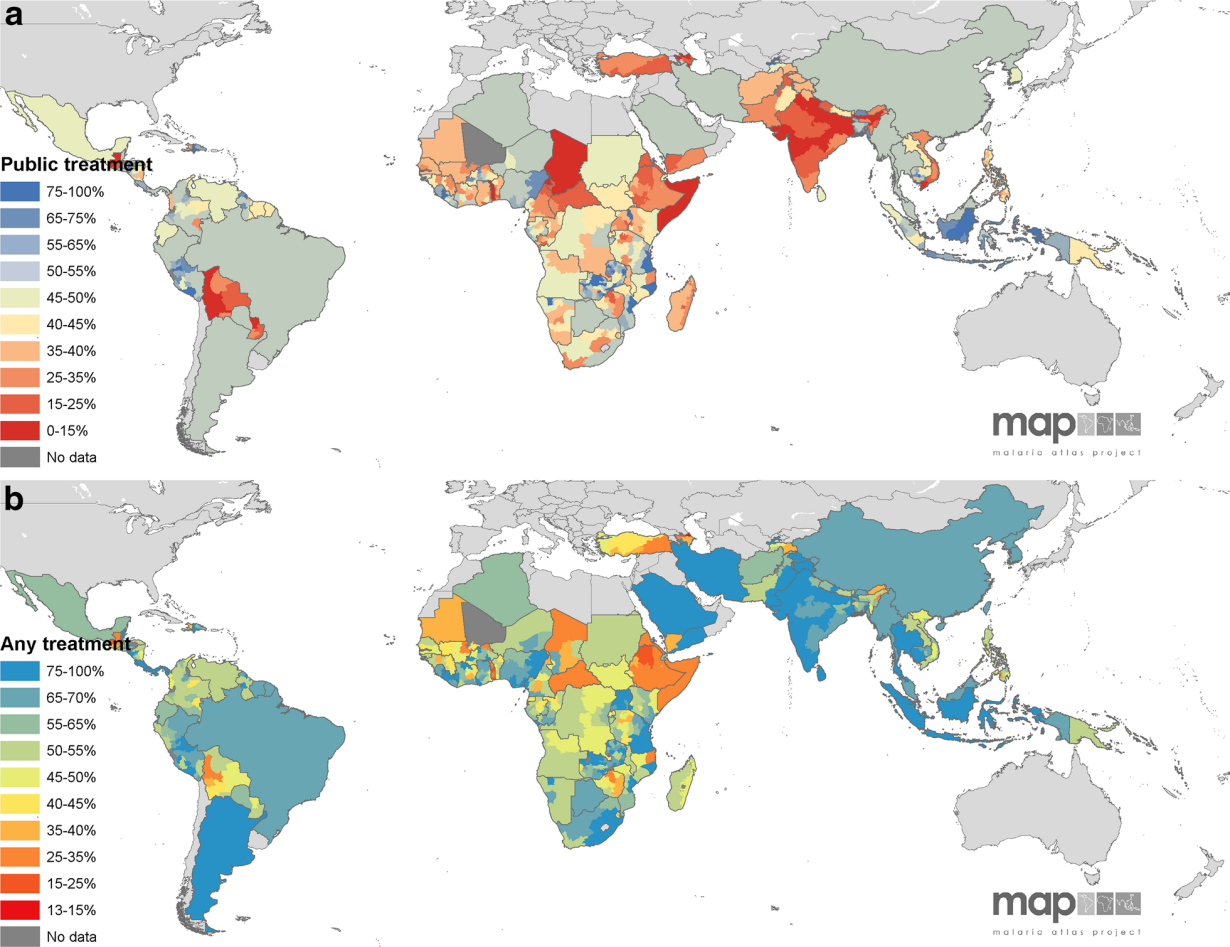
Observed and predicted treatment-seeking proportions. The observed treatment-seeking values in the regions shown in Fig. 1 are mapped along with the national-level predicted values for the proportions seeking **a** government/public treatment and **b** any treatment. Treatment-seeking rates are shown from *red* (low access) to *blue* (high). *Dark grey* areas are those with no data and *light grey* regions are at no malaria risk

The 19 predicted and five assigned national treatment-seeking estimates were combined with those countries that already had data available. Figure 3 shows maps for both the proportion seeking government treatment (Panel a) and those seeking any treatment (Panel b). Note that for both regional comparisons and mapping, priority was given to DHS surveys if both a MICS and DHS survey were reported within the same four years. DHS data are georeferenced which allows for sub-national mapping and are thus considered more representative and facilitate more extensive potential downstream analyses. The maps illustrated the patterns exhibited in the country-level (Additional file 2) and regional estimates (Table 2). Government-based treatment was shown to not be well accessed in large parts of SEARO, EMRO, AFRO and small patches of PAHO. The map of treatment rates from any sector highlighted areas where treatment of was low overall.

## Discussion

Information regarding the treatment-seeking behaviours of fever cases in MECs is essential to assessing the feasibility and success of malaria control and elimination programs. Malaria is a treatable disease and while effective therapies exist, population health-seeking behaviours may limit the extent to which they are utilized. Understanding care-seeking rates in MECs also helps to quantify the scale of the malaria burden. Routine surveillance data are used to measure the burden of clinical disease and only those cases who seek care at a government-based facility are likely to be included in regional estimates. Quantification of the proportion of cases that are severe enough to seek care (clinical), but are missed from passive surveillance, is therefore necessary to more accurately estimate ‘true’ case numbers.

Data obtained from national surveys (DHS and MICS) could be assembled for the majority of the MECs (78 %, *n* = 76). Treatment seeking rates specific to fevers were available from all DHS surveys and MICS 5, while only cough-based treatment rates were available from the earlier MICS surveys. Precedent exists for using care-seeking for respiratory infection as a proxy for fever treatment [14, 25], and this was evidenced by comparable treatment-seeking rates for cough and fever in the observed data gathered here (Additional file 1: Figure S2). Therefore, all available treatment-seeking data were used to inform the model. However, because fever is a primary symptom of malaria and because DHS data are georeferenced and could be mapped sub-nationally, DHS data were given precedence when generating final mapped outputs and summary estimates of the data gathered. Sub-national predictions and mapping of the treatment-seeking outcomes using cluster-level data to produce smooth surfaces like those produced by the Malaria Atlas Project for prevalence was also explored [2, 53, 54]. However, there were not sufficient covariate data available at smooth resolutions at the time of this analysis to support this. The quantity and quality of higher resolution sub-national covariate data that can be used in geostatistical analyses continue to improve with time and there may be greater potential for this type of analysis in the future [55].

Gathering treatment-seeking data for all MECs was the primary aim of this analysis, with the intent to also show sub-national rates where possible. Data that corresponded to the covariate variables identified in the literature review of key factors determining treatment seeking behaviour were readily available from the World Bank and produced models to show that government treatment-seeking and any treatment-seeking could be predicted at the national level from a limited set of covariate variables: year, WHO region, percent of pregnant women that receive prenatal care, immunization rates, primary education completion rate, GDP growth, and national health expenditure (public and total). The percentage of women receiving prenatal care was a strong indicator of fever treatment-seeking that likely drove the low model error values observed. The differing drivers of seeking care from either source were evidenced through the difference in the best models for each treatment-seeking outcome. Government-based treatment seeking was predicted primarily by other health-seeking indicators such as the childhood immunization (DPT) and prenatal care. Access to any treatment, on the other hand, was also influenced by country wealth and education. Educated individuals in more economically stable countries are therefore more likely to spend money on health care outside of the government system.

From the assembled observed data and modelled missing data, there emerged geographic patterns in both the outcomes and the certainty of the predictions. There were areas with low access or use of public treatment facilities in all regions of the malaria-endemic world. Figure 3 highlights areas such as Central Africa and Indian sub-continent. Accessing treatment of any kind was inherently higher in all countries because government facilities are included in that metric. However, the any treatment data revealed that treatment-seeking in some endemic areas, such as India, Pakistan and Afghanistan, was largely pursued outside the public sector. The CI ranges (Fig. 2) show that outcomes were well predicted in the Americas, but less so in Asia and least accurate in the Eastern Mediterranean countries. This implies that models and indicator variables were better suited to the Central and South American countries. Future predictions of this nature may be improved by including additional covariates or, following further research into treatment-seeking indicators, parameters that are tailored to each region.

The comparison of the two treatment-seeking outcomes and the measures of uncertainty provide valuable information as countries define control and elimination goals. The predicted measurements resulted in the greatest uncertainty, and signal the need for treatment-seeking to be formally assessed in those regions. Most notably, this data assembly and analysis reveals parts of the malaria endemic world where treatment is primarily sought outside of government programs. In light of concerns regarding the spread of antimalarial resistance [56, 57], it is essential for countries to ensure that cases are being diagnosed and treated properly using approved and legitimate drugs [58]. If treatment is most commonly sought outside of government-based facilities, control programs must consider how best to monitor treatment safety and efficacy as well as numbers of cases presenting for treatment.

## Conclusion

Information on treatment-seeking behaviours in malaria endemic countries can be readily assembled from national survey data. Where data on treatment-seeking behaviours were not available from national surveys, modelling techniques using freely available data were applied to fill data gaps. Both the results and methods presented have potential application beyond those described here and may inform the control and burden of other febrile diseases. However, in this context, data on treatment-seeking for fever are essential to understanding the efficacy with which malaria cases are treated and detected. Gathering and visualizing these data for all MECs, sub-nationally when possible, is of use to estimate the burden in areas of low endemicity where passive surveillance is the primary tool through which cases are monitored. These results will facilitate downstream efforts to produce a hybridized burden estimation approach that employs both surveillance-based and cartographic techniques in an effort to more accurately quantify the global burden of falciparum and vivax malarias and provide immediate feedback regarding parts of the malaria endemic world where treatment for malaria is not readily accessed or is more commonly sought beyond the government or control programme sectors.

## Additional files

10.1186/s12936-015-1048-x Supplementary information for: Gap filling country-level knowledge of treatment-seeking behaviour in malaria-endemic countries. Description: Supplementary methods, additional figures and tables regarding model development and validation are shown here.
10.1186/s12936-015-1048-x Title: National level treatment-seeking and indicator data for malaria-endemic countries. Description: Treatment-seeking data extracted from national surveys and national-level indicator data from the World Bank are provided here along with the predicted estimates derived from the models described in the main text and Additional file 1.
