# Supplementary material for: Treatment-seeking rates in malaria endemic countries
Source: Malar J. 2016 Jan 11;15:20. doi: 10.1186/s12936-015-1048-x (PMC4709965; doi:10.1186/s12936-015-1048-x)
Supplement: Supplementary file 1 — 10.1186/s12936-015-1048-x Supplementary information for: Gap filling country-level knowledge of treatment-seeking behaviour in malaria-endemic countries. Description: Supplementary methods, additional figures and tables regarding model development and validation are shown here. [file 12936_2015_1048_MOESM1_ESM.docx]

**Additional File 1**

**Supplementary information for: Treatment-seeking rates in malaria-endemic countries**

Katherine E. Battle, Donal Bisanzio, Harry S. Gibson, Samir Bhatt, Ewan Cameron, Daniel J Weiss, Bonnie Mappin, Ursula Dalrymple, Rosalind E. Howes, Simon I. Hay, Peter W. Gething

This file contains:

Supplementary methods

Supplementary Table S1

Supplementary Figures S1 and S2

**Supplementary methods**

**Model development**

Two generalized additive mixed models (GAMMs) were developed to predict the proportion of people that sought treatment from (i) a facility covered by the government reporting system (MOD1), and, (ii) any medical care provider (MOD2) [[46](#_ENREF_46),[47](#_ENREF_47)]. Where multiple surveys were used from the same country, this was accounted for by adding a country-level random effect term. Both survey year and the WHO region the country is in were included as fixed effect terms, along with the suite of country-level covariates.

The Spearman’s rank correlation test was used to investigate the collinearity among the covariates and several indicators were excluded. The full list of covariates examined was as follows: access to electricity, gross domestic product (GDP; current US$), GDP per capita, GDP growth (annual %), gross national income (GNI) per capita (current US$), total health expenditure (% of GDP), public health expenditure (% of total), primary education completion rate (% of relevant age group), rural population (% of total population), the number of community health workers per 1,000 people, the number of nurses and midwives per 1,000 population, the percentage of pregnant women receiving prenatal care and the percentage of children aged 12-23 months who were immunized against diphtheria, pertussis and tetanus (DPT). The number of community health workers per population was excluded first as this data was not available for many of the malaria endemic countries (MECs). Next, access to electricity, which was correlated with most other variables except for pregnant women receiving care and total health expenditure, was dropped. There was correlation among all of the economic variables, so all of the GDP and GNI variables except GDP growth were excluded, as the latter was the least correlated with the other variables. Literacy and primary education completion rates were highly correlated and the latter was retained since it had been directly referenced in the literature search of care-seeking indicators.

Following this covariate reduction, the final set of covariates in the full model was as follows: year, region, GDP growth, health expenditure, prenatal care rates, primary education, DPT immunization rates, nurses and midwives per population and proportion rural population. For predicting treatment-seeking at government facilities, public health expenditure data were used, whereas for any treatment, total health expenditure data were applied. Otherwise, the initial covariate selections were the same between the two models. Time was added a smoothed effect because the effect of the year on treatment-seeking was shown to have a non-linear effect as shown in Figure S1.

Formally, the GAMM was defined as:

*y_care_= β_0_ + β_1 *_ f(Year) + β_2 *_ Region + β_3_*GDP Growth + β_4_*Health Expenditure + β_5_ * Pregnant Women Care + β_6_ * Primary Completion + β_7_ * Rural + β_8_ * DPT + β_9_ * Nurses and midwives + f_rand_(Country)*

Where *y_care_* is the percentage of the country’s population seeking health care for fever (or cough depending on the survey source). *β_1_ * f(Year)* is the non-linear effect of the survey year*, β_2_ *R*egion is the effect of the WHO region, *β3*GDP Growth* is the effect of the percentage annual growth of GDP, *β_4_*Health Expenditure* is percentage of the total country GDP that was spent in public health sector (for MOD1) or any health sector (for MOD2), *β_5_ * Pregnant Women Care* is the percentage of pregnant women who receive prenatal care, *β_6_ * Primary Completion* is the country intake ratio to the last grade of primary education (the number of individuals starting last grade of primary education regardless of age, divided by the population of the entrance age for the last grade of primary education), *β_7_ * Rural* is the percentage of the total population living in rural areas, *β_8_ * DPT* is rate of DPT immunization in children one to two years of age, *β_9_ * Nurses and midwives* is the effect of the number of nurses and midwives per 1,000 people and *f_rand_(Country)* is the country random effect. MOD1 and MOD2 were performed adopting the Gaussian distribution as a link family.

**Model selection**

Model selection was performed using a multi-model selection approach [[49](#_ENREF_49),[50](#_ENREF_50)]. Candidate models with different combinations of the selected covariates were compared based on their model fit, assessed by the Akaike Information Criterion (AIC). The model with the lowest AIC was considered to the best model and the other candidate models that had a difference in AIC (ΔAIC) <2 were also selected to be included in the list of best models successively used to obtain an average model [[50](#_ENREF_50),[51](#_ENREF_51)]. The Akaike weight (ω_i_) from each model was used to assess relative variable importance [[50](#_ENREF_50),[51](#_ENREF_51)]. *β_1_ s(Year)* and *β_2_(Region)* were included in every candidate model because both variables were considered essential to address the spatial-temporal component of the model. Predicted treatment-seeking outcomes for each country with missing survey data were then obtained from the average models.

**Model validation**

To incorporate the uncertainty of the observed treatment-seeking rates, the average models were run 1000 times sampling from the range of the 95% confidence intervals (CI) of the treatment-seeking rates obtained from the DHS and MICS surveys. The mean, upper and lower CI values of the predicted estimates were calculated from these simulations.

The predictive accuracy of the average models was assessed through an out-of-sample model validation. The data used to fit the candidate models were randomly split into 70 and 30%, to serve as the training and test dataset, respectively. The average models for both treatment-seeking outcomes were then fitted to the training set and used to predict the test data. The predicted and observed treatment-seeking values of the test dataset were then used to calculate the root mean square error (RSME) for each model.

**Table S1. Average GAMM coefficients and 95% CIs for each region.** Two generalized additive mixed models (GAMMs) were fit to treatment-seeking data from 76 countries to predict the proportion of those that sought either public or any treatment in 22 countries lacking data. The two best candidate models for government treatment seeking and the two best for any treatment seeking were averaged to generate the predicted values. Geographic regions, defined by WHO region, were a fixed effect in both model sets: Region of the Americas (PAHO), Eastern Mediterranean Region (EMRO), European Region (EURO), Southeast Asia Region (SEARO), and Western Pacific Region (WPRO). Countries in the African region were separated into the sub-African regions reported in the World Malaria Report: West Africa (AFRO-W), Central Africa (AFRO-C), East Africa, and high-transmission areas in Southern Africa (AFRO-E), and low-transmission Southern African countries (AFRO-S)

| **Region** | **AFRO-C** | **AFRO-E** | **AFRO-S** | **AFRO-W** | **EMRO** | **EURO** | **PAHO** | **SEARO** | **WPRO** |
| --- | --- | --- | --- | --- | --- | --- | --- | --- | --- |
| **N** | 57 | 44 | 12 | 23 | 8 | 5 | 46 | 25 | 16 |
| **Countries** | 16 | 11 | 4 | 8 | 6 | 3 | 16 | 7 | 5 |
| **Public** |  |  |  |  |  |  |  |  |  |
| **Value** | -12.15 | -2.12 | 8.85 | -0.60 | 6.34 | 3.43 | 1.08 | 1.82 | 4.79 |
| **Low95%** | -0.24 | -0.09 | -0.03 | 0.08 | -0.06 | -0.10 | -0.06 | -0.07 | -0.04 |
| **High95%** | 0.001 | 0.05 | 0.21 | 0.07 | 0.18 | 0.65 | 0.08 | 0.10 | 0.14 |
| **p-value** | 0.05 | 0.53 | 0.15 | 0.87 | 0.30 | 0.60 | 0.76 | 0.68 | 0.31 |
| **Any** |  |  |  |  |  |  |  |  |  |
| **Value** | 10.69 | 1.25 | 5.64 | -1.38 | 19.31 | -15.20 | 1.70 | 17.49 | 7.31 |
| **Low95%** | -0.04 | -0.06 | -0.07 | -0.10 | 0.07 | -0.30 | -0.06 | 0.08 | -0.03 |
| **High95%** | 0.25 | 0.08 | 0.18 | 0.07 | 0.32 | -0.01 | 0.09 | 0.27 | 0.17 |
| **p-value** | 0.14 | 0.73 | 0.38 | 0.73 | 0.002 | 0.04 | 0.66 | <0.001 | 0.16 |

**Figure S1 The years and countries with available national survey data on treatment-seeking behaviour.**

**
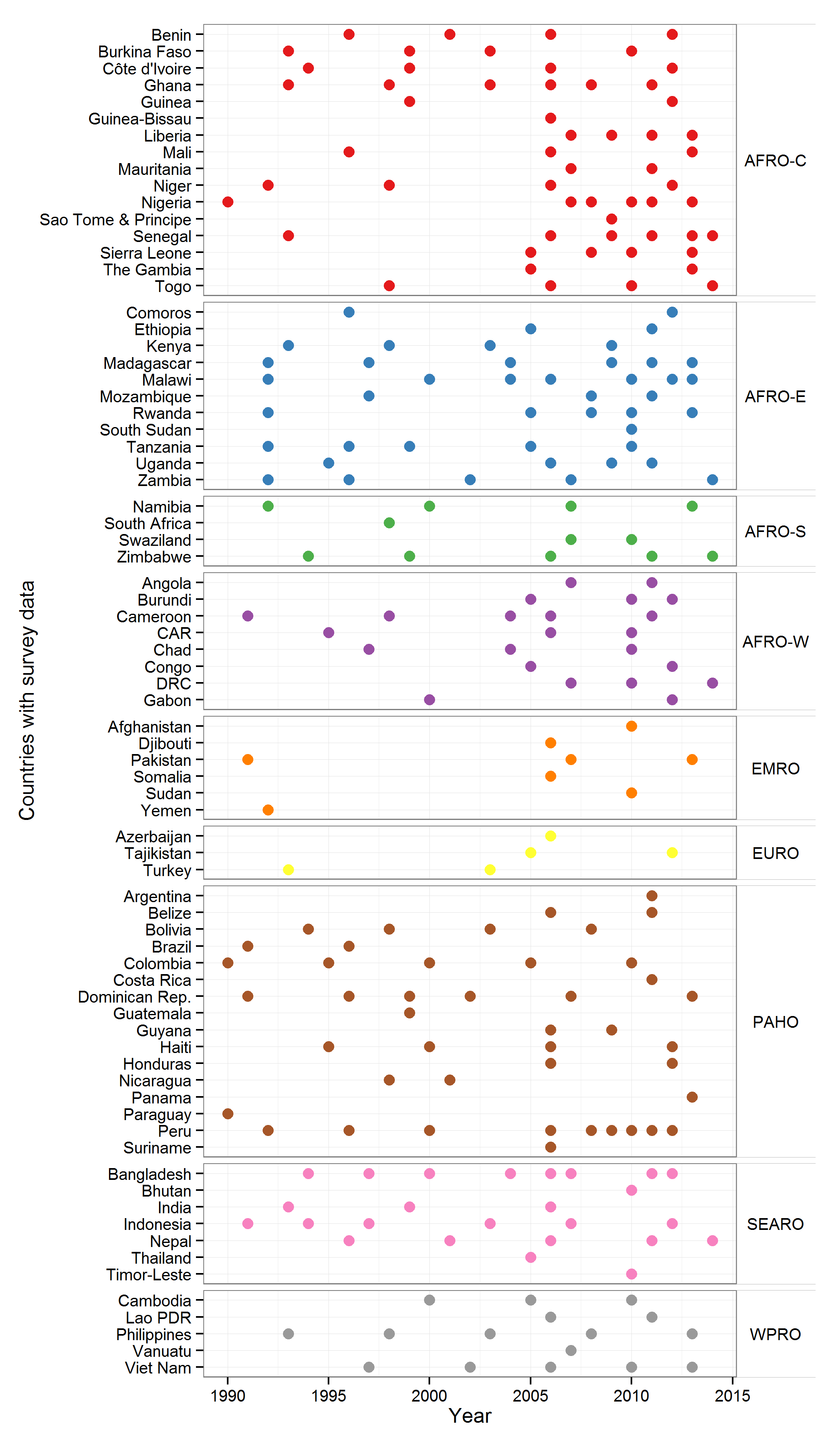
**

**Figure S2 Observed treatment-seeking rates by source.** DHS and MICS 5 surveys assessed treatment-seeking for fever, while MICS 3 and 4 surveys addressed treatment-seeking for cough. The top panel shows the percentage of patients seeking treatment from public or government facilities, while the bottom shows treatment seeking rates from any facility type.

**
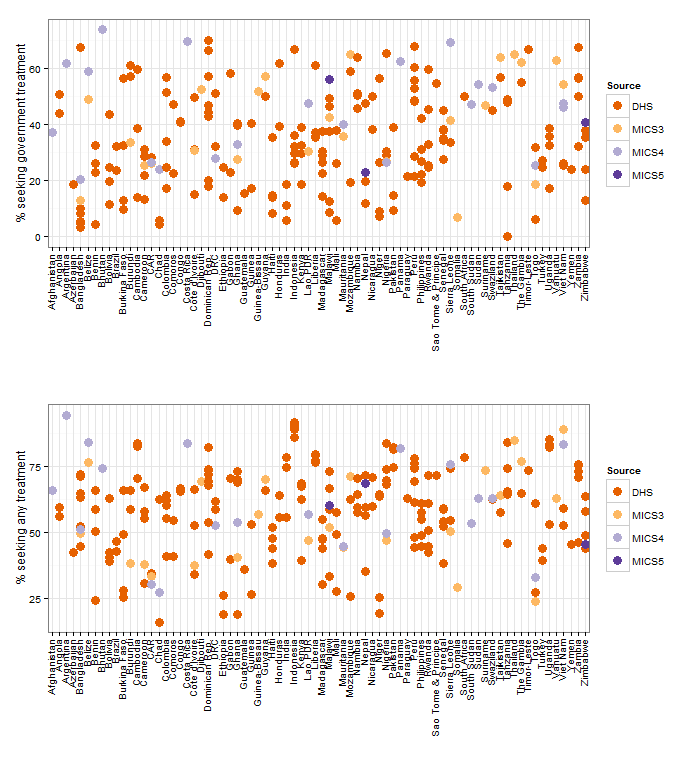
**

**Figure S3 Generalized additive mixed model partial residual smooth plots for year.** The solid lines show the predicted value of treatment-seeking as a function of time for MOD1 (public treatment-seeking, top) and MOD 2 (any treatment-seeking, bottom).


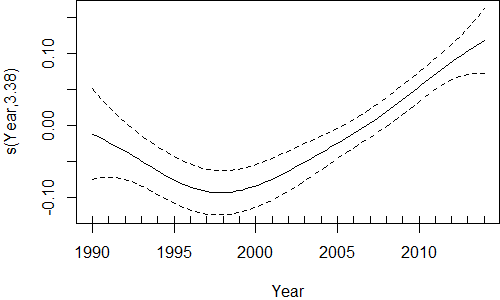


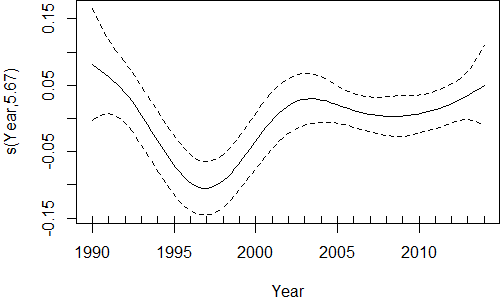


The figures below illustrate how well the models fit the data. First, plots in Figure S1 from the model validation show the predicted values (coloured by region) and 95% CI ranges relative to the observed values (in black) The plots of the residuals versus the fitted values in Figure S2 show homogeneity and the residual histogram plots are normally distributed. Goodness of fit is also illustrated by the plots of fitted versus observed values in Figure S3.

**Figure S4 Observed versus fitted values.** Following model validation, the predicted values for the test dataset are shown in colour with 95% CI error bars for both public (top) and any treatment-seeking (bottom). The observed values for the same data are in black.


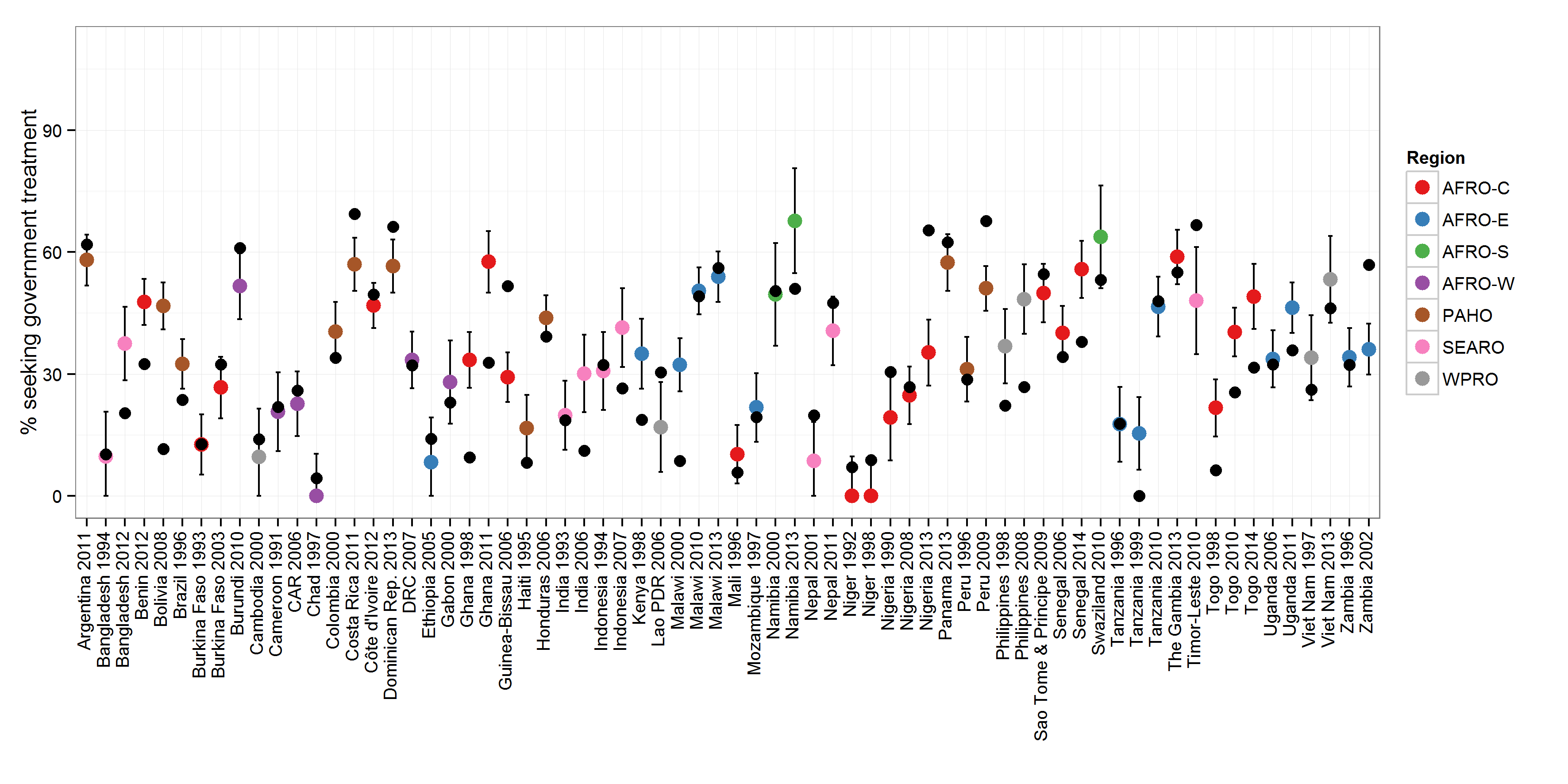


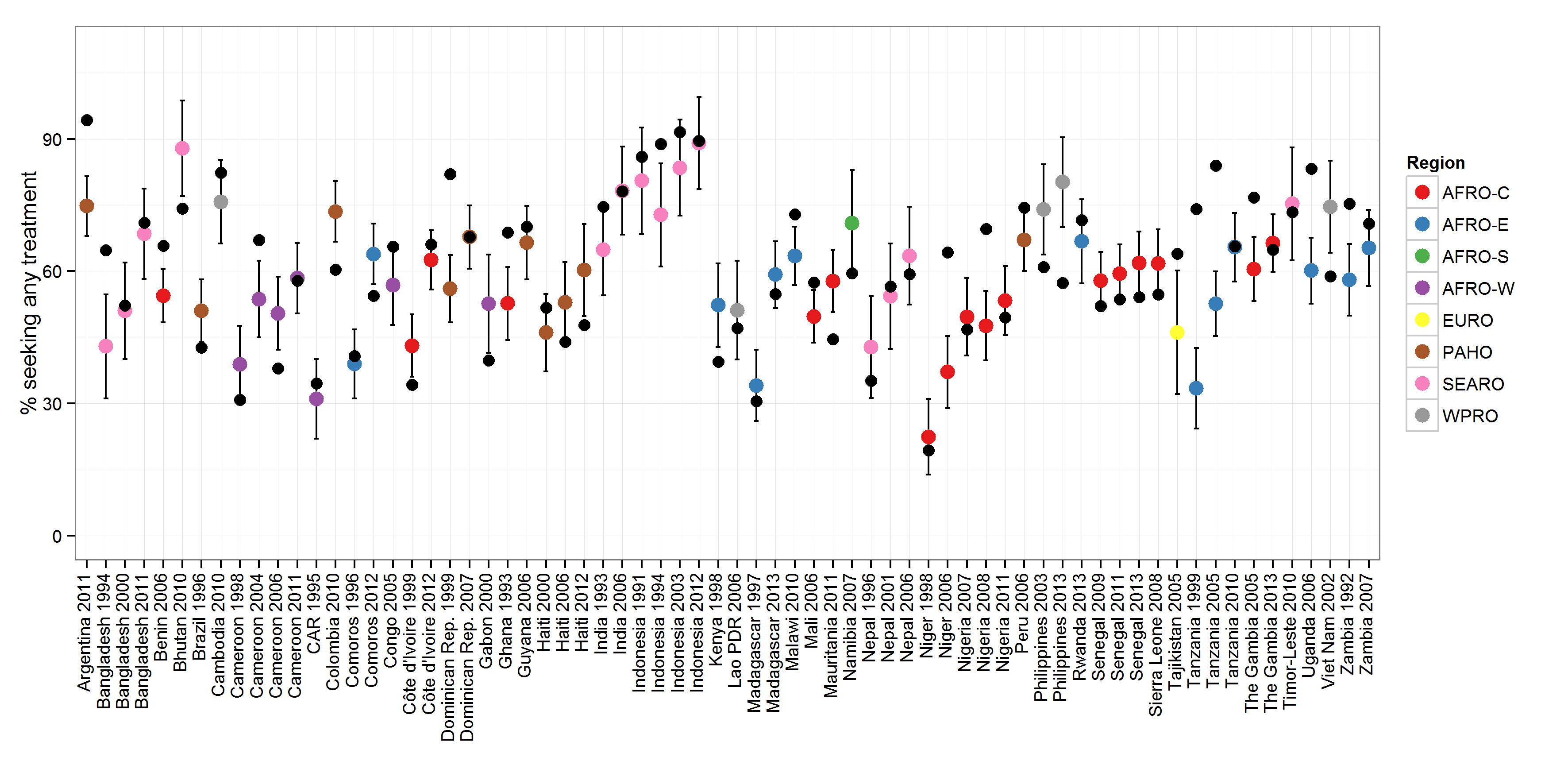


**Figure S5 Model validation plots.** The top rows show plots of residuals versus the fitted values for public (left) and any (right) treatment-seeking. The bottom rows are histograms of the residuals.


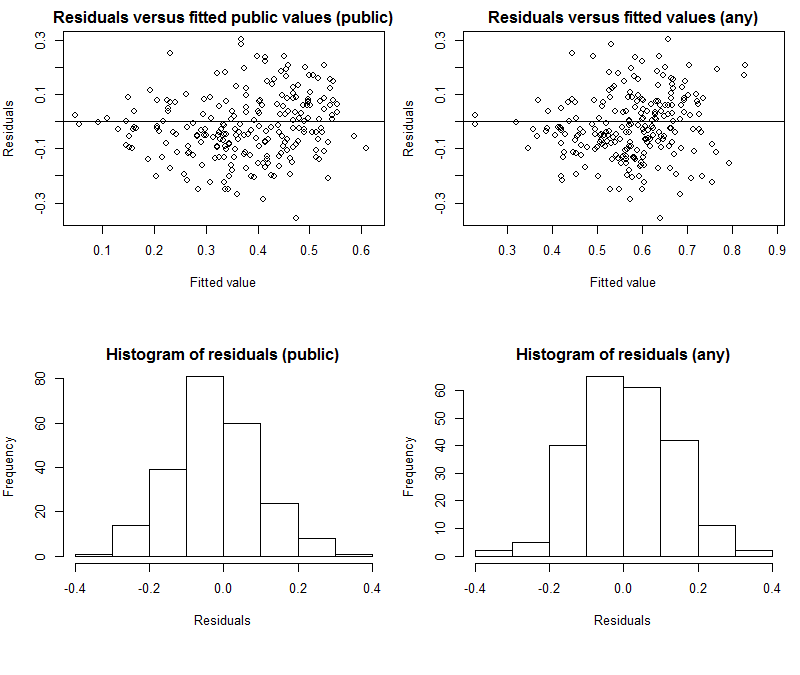


**Figure S6 Fitted versus observed.** The predicted data is plotted against the observed records for those countries and years that had treatment-seeking data available.

**
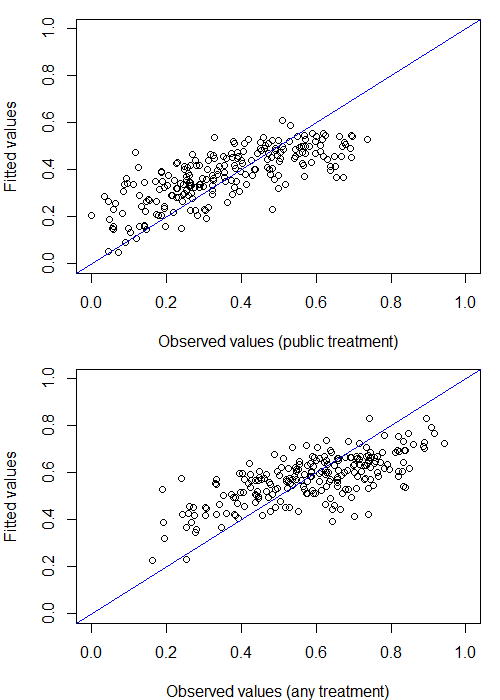
**
